# Propagation, detection and correction of errors using the sequence database network

**DOI:** 10.1093/bib/bbac416

**Published:** 2022-10-20

**Authors:** Benjamin Goudey, Nicholas Geard, Karin Verspoor, Justin Zobel

**Affiliations:** School of Computing and Information Systems, University of Melbourne Parkville, Victoria, 3010; School of Computing and Information Systems, University of Melbourne Parkville, Victoria, 3010; School of Computing Technologies, RMIT University Melbourne, Victoria, 3000; School of Computing and Information Systems, University of Melbourne Parkville, Victoria, 3010

**Keywords:** Sequence, Propagation, Network analysis, Error detection, Annotations

## Abstract

Nucleotide and protein sequences stored in public databases are the cornerstone of many bioinformatics analyses. The records containing these sequences are prone to a wide range of errors, including incorrect functional annotation, sequence contamination and taxonomic misclassification. One source of information that can help to detect errors are the strong interdependency between records. Novel sequences in one database draw their annotations from existing records, may generate new records in multiple other locations and will have varying degrees of similarity with existing records across a range of attributes. A network perspective of these relationships between sequence records, within and across databases, offers new opportunities to detect—or even correct—erroneous entries and more broadly to make inferences about record quality. Here, we describe this novel perspective of sequence database records as a rich network, which we call the *sequence database network*, and illustrate the opportunities this perspective offers for quantification of database quality and detection of spurious entries. We provide an overview of the relevant databases and describe how the interdependencies between sequence records across these databases can be exploited by network analyses. We review the process of sequence annotation and provide a classification of sources of error, highlighting propagation as a major source. We illustrate the value of a network perspective through three case studies that use network analysis to detect errors, and explore the quality and quantity of critical relationships that would inform such network analyses. This systematic description of a network perspective of sequence database records provides a novel direction to combat the proliferation of errors within these critical bioinformatics resources.

## Introduction

Databases that catalogue genetic and protein sequences have been a cornerstone of bioinformatics analyses for over 30 years [1]. Records in these databases correspond to genetic sequences and corresponding metadata and provide details about the source of sequence, the submitters or annotations of the sequence itself. Databases such as GenBank [2], RefSeq [3] and Pfam [4] pre-date the Human Genome Project, with initial releases consisting of a few hundred short sequences, manually curated by a team of expert annotators [5]. Since that time, these databases have grown at extraordinary rates, with GenBank now holding over 2.5 billion sequences covering 504 000 formally described species [6]. This growth has outstripped the ability of manual curation to ensure data quality, leading to a near-complete reliance on the automated tools for annotation of sequence records [7, 8].

Despite the widespread use of sequence databases, there is a growing body of evidence that sequence databases contain substantial levels of spurious information such as duplication [9], contamination of sequence data [10, 11], incorrect annotation of protein function [12–14] and spelling mistakes in protein descriptions [15, 16]. Errors of these kinds are challenging to detect because the distinction between natural genetic variation and spurious data is often unclear. The rapid growth of sequence databases has led to an increasing reliance on automated record annotation, which however has been shown to significantly increase the likelihood of errors across a range of record annotations [17] that in turn can be propagated to future records [15]. Such erroneous records have led to serious mistakes in downstream analyses, including incorrect identification of pathogens due to mislabelled sequences [11], incorrect conclusions about genome evolution [18] and spurious conclusions about horizontal gene transfer [19–22].

**Figure 1:**
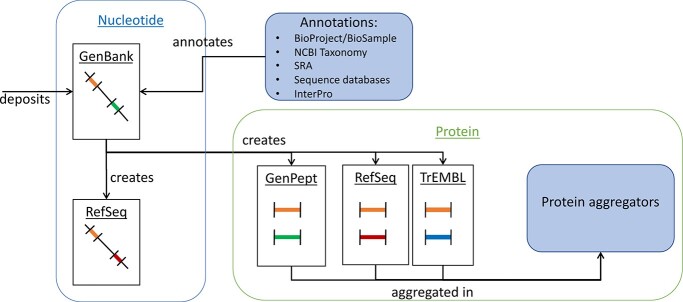
Example of a record and annotation propagation. Here, we consider a nucleotide sequence (shown as a diagonal line) that is uploaded to GenBank, where annotations of proteins (shown as coloured bars) are drawn from a wide range of databases, including from other records in GenBank. A further record for this sequence is created in RefSeq, with corresponding protein records (shown as coloured horizontal lines) created in the databases GenPept, RefSeq and TrEMBL, where the different colours indicate different protein annotations. Furthermore, all records may be used to annotate records deposited in the future.

While many techniques have been proposed to identify errors that affect sequence records, most approaches to date do not fully exploit the highly connected nature of sequence records and databases. Consider the hypothetical example in Figure 1, illustrating a novel sequence that is submitted to the nucleotide sequence database GenBank [2].

- Metadata for this record may explicitly point to upstream project and sample records in BioProject [23] and BioSample [23] databases; it will be annotated with taxonomic information from the NCBI Taxonomy database [24], and may even be linked back to raw read or assembly data in the Sequence Read Archive (SRA) [25] or NCBI Assembly [26].
- The newly submitted record may have its genomic features annotated based on protein feature databases that form the Interpro collection [27] or from existing nucleotide and protein records which may be similar in sequence content, taxonomy classification or protein features. These annotation relationships may be explicitly recorded or may be implicit, requiring some inference to quantify the relationship.
- Identified protein-coding regions will generate novel protein records in GenPept [28], with further proteins potentially identified using TrEMBL [5].
- If the submitted nucleotide record is deemed to be non-redundant and of high quality, it may be re-annotated in RefSeq [3], which may lead to further protein records.
- Any identified proteins will be indexed by protein aggregator databases such as NCBI Protein and UniParc.
- In some circumstances, these records may contribute to new protein families or features recorded as part of the Interpro collection.
- Finally, the novel record itself may also be used to annotate other sequence records that are deposited in the future.

Beyond these explicit relationships, all nucleotide and protein records have implicit relationships to all other records based on sequence, taxonomic and functionality similarity, which can be derived using a variety of different measures.

The example above highlights that a single sequence record uses, creates and relates to a range of records within and across databases [29]. We can view these relationships between records as a highly connected network that spans a range of sequence databases which we call the *sequence database network*, in which each real-world item is represented in different ways and different contexts in different databases, but the representations are linked to each other. The interconnected nature of the sequence database network provides the potential to amplify the impact of errors in sequence records but also offers new opportunities to uncover errors and quantify database quality.

In this paper, we describe this network and how it can be used to understand and improve data quality of sequence databases. A *network perspective* in which each record is regarded as a single entity with multiple relationships to representations and sources of annotation can, as we show, provide the basis of new, rich techniques for management of sequence records.

We provide three case studies that highlight different strengths of this network perspective. The first case study frames existing approaches to error detection as forms of network analyses and highlights how our network perspective provides opportunities for further methodological development. The second relates to the role of implicit information in the network, focusing on estimates of annotation confidence that are currently lacking for most annotations and are critical for error analysis. The third concerns the quality of recorded annotation provenance, information that is key for forming a network from annotations and for understanding how errors propagate across the network.

Combined with our reflection on database connectivity, this review makes a clear case that the network perspective is a valuable approach to the analysis of sequence records and thus the review highlights potential extensions to existing methods and opportunities for the application of novel techniques.

## An overview of sequence databases

Before providing a more comprehensive description of how we define the sequence database network and the impacts this may have, we need to define the types of databases and relationships between constituent records that form the network.

**Figure 2:**
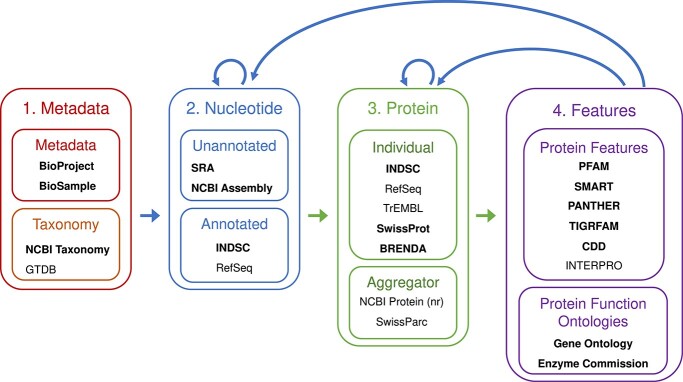
Overview of the four types of data reviewed in this work: the arrows between the databases correspond to information flow, with the blue arrows indicating propagation of annotations or metadata from the source database to a new record in the target database, while the green arrows indicate that a new record in the source database could lead to new records in the target database. Within the four database collections, further sub-categorization of the databases is shown. The bold text indicates that users can directly upload to a given database.

A wide range of databases have been created to store and catalogue information related to sequence records. We identify four main classes of databases, with a partial overview of key databases as shown in Figure 2:

- **Metadata:** A disparate set of databases hold information that forms a key part of sequence record metadata, including information about the initiative or consortium that generated the data (BioProject [23]) or the type of sample from which a sequence was generated (BioSample [23]). This category also includes taxonomic databases (primarily NCBI Taxonomy [24]), which have a critical role as a source of annotations for downstream sequences.
- **Nucleotide sequences:** Nucleotide databases can be categorized into two classes. Unannotated sequences including raw read sequences (SRA) and annotated assemblies (NCBI Assembly) have been stored since 2007 and 2011, respectively. In contrast, databases related to annotated nucleotide sequences make up the majority of records in the genomic sequence database with International Nucleotide Sequence Database Collaboration (INSDC) databases [30] (including NCBI GenBank [2], EMBL European Nucleotide Archive (ENA) [31] and the DNA Data Bank of Japan (DDBJ) [32]) now containing 1.6 billion nucleotide records. Annotated sequences are typically submitted to the archival INSDC databases, that is, sequences remain owned by submitters [33], and are annotated using a wide variety of pipelines that vary greatly in their accuracy. A subset of “high-quality, non-redundant” [3] sequences are then reannotated and stored in the RefSeq database. These records are owned by the INSDC consortia, allowing them to be updated if errors are detected.
- **Protein sequences:** There is a large variety of protein sequence databases, which differ in the criteria and source data used to record protein sequences. INSDC databases provide records for all proteins annotated in stored nucleotide sequences based on user-specified annotations. As such, any user-submitted protein sequence requires a record containing the corresponding nucleotide coding sequence. A single nucleotide record in an INSDC database will often produce many protein records. A similar process exists for RefSeq databases, albeit using a consistent annotation pipeline. UniProtKB/TrEMBL [34] is an uncurated database with protein sequences that are automatically inferred from all INSDC nucleotide databases, RefSeq, and a range of other sources. In contrast, UniProtKB/SwissProt [34] contains protein records that follow strict biocuration guidelines and require strong experimental evidence, constraining the number of available records but giving greater assurance about the quality of the functional information. Others are more specific, with for example BRENDA [35] containing only enzymes, that is, proteins that act as biological catalysts.
- **Sequence feature databases:** Databases related to specific sequence features are critical for the annotation of new sequences. One key group of databases focus on cataloguing nucleotide and genomic features, including Pfam [4], SMART [36], TIGRFAMs [37], PANTHER [38] and CDD [39]. These databases are aggregated in collections such as InterPro [27] and provide the basis for large collections of hidden Markov Models (HMMs) that are used to annotate new proteins [40, 41]. A second group of databases, primarily Gene Ontology [42, 43] and Enzyme Commission [44], provide specific labels that describe the biological function of proteins and enzymes, respectively.

Information from the metadata databases is used to annotate nucleotide and protein sequences. Nucleotide sequences are used to generate many of the recorded protein sequences, and novel nucleotide and protein sequences are used to improve the sequence feature databases, leading to implicit relationships based on these dependencies. Existing sequence records and information in the sequence feature databases are used to annotate new genomic and protein sequences, forming feedback loops. The network contains further complexities due to the ability of users to enter data independently in many databases, which may lead to variation in the quality of annotations or potential duplication of sequences across databases [9]. Moreover, the curation strategies of the different databases vary from fully automated to entirely manual, not only impacting the reliability of record metadata, but also changing the rate at which information flows between databases. Records in the network may also change over time due to curator updates or changes in database criteria. These changes are ideally (but not always) propagated to all dependent records. This set of interconnected, mutually informing data provides a rich source of information that can be used to derive inferences about sequence records across these key databases.

The different nucleotide and protein sequence databases vary significantly in their intent, a factor that is reflected in their curation processes and ultimately the information content of the records themselves. INSDC databases are archival and serve as a primary database of all nucleotide sequences, with GenPept and EMBL Coding Sequences (CDS) recording user-derived translated protein sequences. In these databases there is no attempt to remove redundant sequences, all annotations are by the submitter and corrections can only be made by the submitter.

In contrast, the aim of RefSeq is to provide high-quality annotations for a subset of organisms that have strong reference genomes and are of broad interest to the community. RefSeq entries are derived from INSDC sequences, re-annotating them using custom pipelines [40, 41]. Redundancy is removed, with sequences from multiple INSDC records merged to form a single composite record. In UniProtKB/Swiss-Prot, the majority of protein records are also derived from INSDC sequences. A subset of protein-coding sequences are re-annotated and manually validated against experimental evidence in public databases and biomedical literature. These proteins are highly non-redundant and contain a wider set of database cross-references than observed in GenBank or RefSeq. As each database’s curation varies, the same nucleotide sequence may lead to different protein records in each database. While the degree of consistency in annotations across databases may be a further source of information to the user about annotation reliability, there has been relatively little study of database consistency [45].

## Defining the sequence database network

Formally, we define a *sequence record network* as a set of individual sequence records, drawn from one or more databases, in which each record is represented as a node, and relationships between two records are represented as an edge between two nodes. Nodes may be connected by different types of relationship:

- *Record generation*, where a new record in a source database leads to creation of new records in a target database. This can be further broken into gene/product relationships, to describe a nucleotide leading to the creation of protein records. Or it could be a parent–child relationship if we are describing the copying of one biological sequence to another database entirely.
- *Annotation propagation*, where metadata or annotations from a source record are propagated to a new target record.
- *Record similarity*, which includes sequence homology, taxonomic similarity and similarity of other metadata, and can be considered between records both within and across databases.

Significantly, these relationships exist not only within a single database but across databases, allowing us to take advantage of differences in curation process or annotation pipelines. While the types of relationships are widely described and many have been utilized in the context of detecting errors, there have been few clear attempts to understand how these relationships may be systematically combined to help elucidate record quality.

The resulting network can be analysed from two angles. The first is by viewing related records as different perspectives of the same (or highly related) entity. In this perspective, we can make use of the rich set of relationships that exist between records to understand the quality of the network and the similarity of annotations across both records and databases. Using our prior knowledge of expected differences, we can make inferences about whether particular annotations are unexpected.

Second, we consider the use of network science techniques on top of this network of records, with the idea of understanding data quality and detecting erroneous entries through the use of outlier detection, community analysis and node/edge classification techniques. These techniques can help us to gain an understanding of patterns of connectivity which may be indicative of errors or may indicate regions of the derived network that require further attention by curators.

## Annotation errors in biological sequence records

The interconnectedness of records within and across sequence databases means that the introduction of spurious information can have far-reaching consequences. To understand how errors can be propagated, either at the time of record creation or through inappropriate propagation of annotations, it is helpful to understand the information contained in a sequence record, how it is derived and how errors may arise.

All biological sequence records consist of two components: the biological sequence itself and corresponding metadata of the sequence. Sequences vary greatly in terms of their length and complexity and in the underlying technologies used to generate them. Metadata varies depending on the record type but often includes the date the record was created, the type of organism from which the sequence was derived and functional annotations of the sequence itself. While most records contain at least some manually curated metadata, there is an increasing amount of metadata that is automatically derived, especially that related to the functional annotations of a sequence [7, 46]. For nucleotide sequences, annotation pipelines typically search for coding genes using a combination of homology to existing proteins or protein families or from the application of HMMs that represent certain types of proteins, falling back to *de novo* annotation if these methods fail [7, 40, 41, 46, 47]. Information about previously annotated genes and proteins is often automatically transferred to unannotated sequences based on these strategies. Similarly, once a protein is derived, its description and protein function codes (Gene Ontology annotations (GOA) or Enzyme Commission (EC) terms) may be inferred using propagation-based approaches [7, 47, 48].

Propagation of annotations has enabled the rapid growth observed in many sequence databases [49]. However, benchmark analysis of propagation pipelines reveals that there can be large differences in the annotations of genomes resulting from different methods [50, 51], which could lead to conflicting annotations within a given database. Such conflicts are also likely to occur in manually curated databases such as UniProt, given the strong inter-annotator disagreement when examining annotations such as protein function [52]. Reliance on propagation of existing annotations also means that, once errors are present within the sequence database network, they have the potential to be propagated to new records [53]. This introduces serious challenges as an error would need to be corrected in multiple records, and often in multiple databases, simultaneously [49].

Automatic annotation based on propagation relies on two assumptions: that annotations in existing records are correct and that we can accurately identify which source records can be used to propagate annotations to a given target record. Therefore, we can classify sources of record error as either errors that first arise in a given record or those that occur due to inappropriate propagation. Errors that originate within a given record can be separated into sequence-based errors, typically derived from steps within the sequence generation process including sequencing error, sequence contamination and assembly errors, while metadata errors encompass all errors within non-sequence information within a record, from sample metadata through to functional annotations.

In contrast, propagation errors are caused by the inappropriate reuse of information from one record to create the content of another. While the relatively strong relationship between sequence and function justifies the propagation of annotations from existing sequences to a new sequence [54], there are limits to how far annotations should be transferred; however, these vary by annotation pipeline and the available records related to a given species. This includes the proliferation of errors to new records, slow or limited propagation of record updates or propagation of annotations to inappropriate target sequences. These types of errors are challenging to detect as there is often no explicit documentation of the source of the propagated information, making it difficult to trace errors to their origin, let alone correct database content [15]. A more detailed analysis is given in Table 1, highlighting common examples of each type of error.

**Table 1:** Sources of errors that affect sequence records, categorized into three broad classes (metadata, sequence and propagation), illustrated by common examples of errors within each of these classes. The column ‘References’ provides references to methods that address the specified issue

	Type	Description	References
Sequence	Assembly errors	Errors in the sequence resulting from poor assembly.	[59, 60]
	Contamination	Errors in the sequence resulting from the introduction of foreign material in the sequencing process	
	Sequencing errors	Sequence errors from errors in the sequencing platform.	[10, 11]
Metadata	Taxonomic misclassification	Incorrect assignment of taxa to a given sequence.	[61–63]
	Functional annotations	Incorrect annotation of the sequence, e.g. incorrectly labelling the function of a protein sequence	[12, 64, 65]
	Annotation boundaries	Inaccurate identification of annotation boundaries, e.g. incorrectly identifying the start or end of a protein in a nucleotide sequence.	[66]
	Data entry	Errors in spelling of metadata fields, e.g. incorrect protein names	[15]
Propagation	Over-prediction	Propagating information from one record to one that is too dissimilar	[13]
	Error propagation	Propagating errors from one record to one another	[15]
	Staleness	Failing to update a record when source record changes	-

It has previously been noted that different types of annotation errors may have more impact than others [55]. We believe that propagation errors have a far greater impact than errors first arising either in metadata or sequences, given the propagation of errors may affect many records deposited over time. While the vast majority of tools for detecting errors in sequence records focus on either errors related to sequencing or metadata, there are fewer studies that explicitly focus on detecting errors stemming from incorrect propagation [13, 14, 56] or even quantifying the confidence in propagated annotations as an indicator of potential error [57, 58]. Explicitly taking a network perspective of sequence records may offer novel approaches for quantifying, tracking and detecting these errors by leveraging the explicit and implicit relationships across records and databases.

We now examine the significance and variety of these issues through three case studies.

### Case study 1: Using network analysis to detect and remove errors

**Proposal:** In this case study, we describe how outlier detection, a common approach to detecting errors in sequence databases, can be usefully reframed from the perspective of sequence database networks.

Detecting any of the errors listed in Table 1 is challenging, in part because there is little gold-standard information as to what the true biological properties of a given sequence are. As such, the majority of methods for detecting errors in sequence records tend to focus on the detection of either specific patterns which typically correspond to the presence of very specific errors or forms of *outlier detection*, that is, searching for records which are unexpectedly different in one or more attribute compared with a neighbouring population. While the former approach is quite constrained, as it requires very specific rules that will only work in particular circumstances, outlier detection can be generalized and has been widely studied [67].

**Figure 3:**
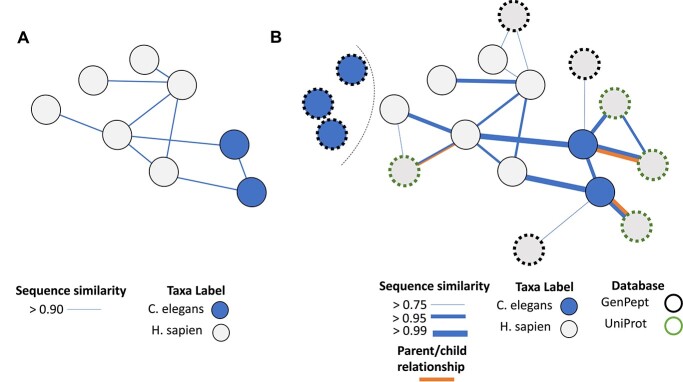
Example of how a network perspective can help inform outlier detection. **(A)** A collection of records, with the grey circles indicating records marked as *Caenorhabditis elegans*, while the blue circles are marked as *Homo sapien*. The lines indicate a sequence similarity between records greater than 95%. **(B)** Expanded network of records. Additional records have been included below a 95% threshold (white, black edge) as well as including records from another database (white, green edge). The size of the edges between node now reflects sequence similarity. Moreover, the orange edges have been included to indicate parent–child relationships. We additionally highlight a collection of blue records as a source of information about what constitutes a typical *Homo sapien* record. This expanded network contains a far greater degree of information but requires more sophisticated methods to integrate this information.

An example of outlier detection for sequence records is shown in Figure 3(a). Here, we show a neighbourhood of eight protein records with links specified between records that have a pairwise sequence identity greater than 95%. Of the eight records, two are misclassified as *Homo sapien*, while the six remaining records are correctly classified as *Caenorhabditis elegans*. Given the strong sequence similarity in the community but high taxonomic distance between these taxonomic labels, it could be reasonable to flag these few *Homo sapien* entries as potentially mislabelled. Identification of sets of records that are similar in terms of one property, but with a small number of records discordant in another property, is a widely applied form of outlier detection and has been used to identify incorrect taxonomic data [61–63], protein function descriptions [12] and GO [64, 65] annotations. However, such approaches typically search for instances where outliers are a small fraction of their nearby community, as low as 1% [61]. The small number of nodes in this community means we have limited evidence to detect misannotated records and hence this form of misclassification may not be detected by standard heuristic approaches.

By reframing the outlier detection approach above in the form of network analysis, we can see opportunities to improve power to detect misannotation by incorporating more available information. The approach described above can be seen as a form of multi-view outlier detection [67], where nodes (records) have a single property (taxonomy in the example) and unweighted edges are based on another property (sequence identity greater than a specified threshold). We can increase our evidence to detect misannotation through additional sources of information on both nodes and edges. Figure 3(b) shows the same nodes but with additional annotations. The community is expanded by making use of the degree of sequence similarity, rather than use of a simple hard threshold. This allows the incorporation of sequence records with identity lower than 95% by giving them less weight in downstream analysis.

Relationships can be expanded to consider similarity in protein function [68] or taxonomic distance via lowest common ancestor [69]. We can also consider additional properties of the records such as quality of the nucleotide sequence or assembly from which the records were derived or the annotated protein function. Finally, we can consider not only the detected community based on sequence similarity, but contrast the records with the community of records based on taxonomic or other record properties, to highlight potential discrepancies.

**Summary:** By expanding outlier detection beyond sequence and taxonomic similarity to also consider network properties, we can approach error detection in richer ways. This network approach to outlier detection can focus on outliers at the level of individual records, small communities of records, or even based on outliers in terms of relationships rather than the records themselves [67]. While these additions add greater complexity to the resulting heuristics for error detection, they also add substantially more contextual information to the task, potentially reducing false positives.

### Case study 2: Interrogating the network to estimate the reliability of annotations

The absence of any indication of annotation confidence is a critical limitation of existing sequence databases. In this case study we examine how a network perspective can help us to quantify the scale of this absence, and also provide an approach to estimating record reliability. We explore *post hoc* methods for computing annotation confidence and highlight its potential role in downstream analyses to detect erroneous annotation.

An implicit assumption of bioinformatics analyses is that sequence annotations in a given record are correct. In practice, the accuracy of annotations is known to be highly variable. One example is seen in the benchmarking of state-of-the-art protein function prediction methods, where prediction accuracy ranges from a maximal F-score of 0.44 to 0.71, depending on the species under consideration [70]. While annotations are always going to be imperfect, indicators of annotation confidence and reliability can be used to enrich downstream analyses. Such metrics are often produced by existing annotation pipelines. However, these indicators of confidence are rarely stored alongside predicted annotations and are instead discarded [71]. This has several implications. The first is that it implies that the derived information is known to be correct. Second, it hinders reliable or informative propagation of annotations to new records, as the propagation is unable to account for the uncertainty of an annotation and its predecessors; noting also that the uncertainty will compound at each step [53]. Finally, annotation confidence could form another important feature that could be integrated into any network analysis of sequence records, upweighting or downweighting properties based on their reliability. Given that there is often no information about the provenance of propagated annotations recorded in most sequence databases, there is a strong need for tools that can infer information related to the reliability of record metadata.

There have been attempts to infer measures of confidence for specific classes of annotations by leveraging cross-database relationships across the sequence database network. One such example is provided by [14], who explore the reliability of enzyme commission (EC) annotations in existing sequences. As part of their study, the authors used approximate sequence identity to the nearest experimentally validated sequence record as a measure of reliability in the assigned EC term. Across sequences from the Archaea, Bacteria and Eukaryota superkingdoms taken from the enzyme sequence database BRENDA, the authors found that almost 20% of enzymes showed less than 25% sequence identity to the nearest experimentally characterized enzyme of the same EC class. This low level of similarity likely indicates low confidence propagation of annotation.

**Figure 4:**
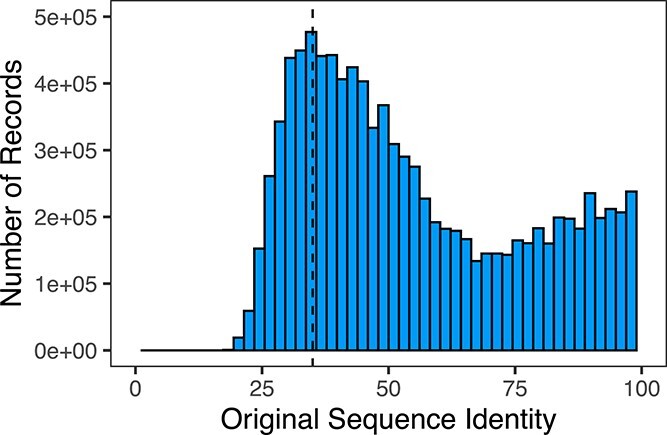
Distribution of sequence identity between bacterial protein records from GenBank that have annotated EC terms and their their nearest experimentally validated sequence in UniProt. The black dashed line highlights poor similarity (below 35%), with approximately 2 million records falling bellow this threshold.

To highlight the potential impact of derived indicators on annotation reliability, we have extended the experiment by [14] to examine the quality of 10.6 million bacterial protein records that are annotated with an EC term. All proteins with a given EC term are compared against protein records with the same EC term but drawn from 11 400 protein sequence records from UniProt that have an Evidence & Conclusion Ontology (ECO) code [72], indicating the EC term was experimentally validated. Using a similar framework based on evaluating sequence similarity [14] but using a more sensitive measure of sequence identity (Supplementary Methods), Figure 4 shows that while the distribution of sequence identity is significantly higher than previously reported, 19% of proteins (2.01 million) show less than 35% sequence identity to an experimentally validated of the same annotated functional class. The limited similarity indicates that we have very little confidence in the functional annotation given to these enzymes and they should be viewed as candidates for re-annotation. This information could be integrated into any downstream network analyses and would also mean these sequences should be downweighted as a source of annotations for new records, given our limited confidence.

**Summary:** Our analysis and the work by [14] highlights how even a simple metric can inform assessment of annotation quality in sequence databases. Parallels can be drawn between this analysis and the outlier detection framework given in Case Study 1. Here, we form communities based on shared enzyme annotations and then searched for outliers where distance to the nearest experimentally validated sequence was below a given threshold (in this case 35%). As with the outlier detection in Case Study 1, inferred confidence scores can be strengthened by incorporating neighbourhoods of records based on sequence identity, function or taxa, which jointly provide more granular information. These types of integrative approaches have been seen in Critical Assessment of Functional Annotation (CAFA) [70], a shared task that seeks to improve automatic protein function prediction. The use of network approaches is increasingly common in the task [73]. These state-of-the-art tools for inferring protein annotation on novel sequences may be adapted to help determine the correctness or reliability of existing annotations, with many tools implicitly making use of cross-database information [74–76]. However, it is unlikely that these *post hoc* approaches to inferring annotation confidence will be perfect and improvements in the initial recording of annotation provenance to capture the confidence metrics from the initial annotation pipeline would provide even strong information in this regard.

### Case study 3: Understanding the quality of the annotation network data

**Proposal:** While some network properties can be inferred, such as annotation confidence, others cannot, such as the source of a record’s annotations. In this case study, we examine how well information about provenance has been recorded, focusing on bacterial records in GenBank.

As the previous case study highlights, the recording of annotation provenance is imperfect across the sequence database network. A lack of provenance within databases is known to have a wide range of impacts on overall database quality [77]. One issue is that of propagation of corrections from a source record to any records that have relied on its annotations. Records with a parent–child relationship in the same database or ecosystem (primarily NCBI and UniProt) are typically updated automatically within the same database release. For records with an annotation source–target relationship, often no such updating is possible as the information about the relationship is not explicitly recorded. As such, the annotations are unable to stay synchronized; errors go uncorrected and hence continue to propagate into downstream databases and analysis. While issues of record updates and the potential for errors to persist within the system have been described [10], there have been few studies explicitly characterizing how spurious annotations propagate through the sequence database network and how feasible it is to correct these annotations.

A study of propagation of erroneous annotations, which also highlighted the difficulty of propagating corrections, is given by [15], who examined the propagation of a specific spelling error (‘Putaitve’ rather than ‘Putative’) in the protein function description of 99 protein records. As these proteins share structural and functional features, it can be inferred that the spelling mistake is a propagated error from automated tools rather than one that has been entered multiple times. The authors identified that many of the early entries in this collection of records have since been corrected, but as the spelling error has been propagated in many proteins it is difficult to remove all instances of the error from the system. At present, the spelling error remains present in 83 records within GenBank and 46 within RefSeq. This relatively innocuous error may not have much practical impact, but does demonstrate how challenging removal of propagated errors can be.

More serious errors can be seen in two recent studies that detected thousands of instances of proteins in the NCBI Protein database that spuriously originated from contaminated sequences [10, 11]. Given the proliferation of the spelling error in the example above, it is likely that these contaminated sequences, now spread across multiple databases, will be used as the source of annotations in the sequence database network even if the original records are corrected or removed.

To highlight the extent to which missing provenance annotation is a problem, we have analysed bacterial sequences in GenBank to understand the extent to which source records are recorded. Such information is available through the use of the ‘inference’ qualifier which has been recorded since 2006 and the ‘note’ qualifier, which can store free text. We also estimate how often corrections are reflected in these annotations. To do this, we focus on the most extreme case, where the records used as the source of annotations have been renamed or removed entirely. These records will have been changed due to a range of reasons including errors in the annotation, poor sequencing or assembly quality or redundancy with existing records. As such, the proportion of ‘dead’ links should reflect a lower bound on the proportion of records where corrections have not been propagated.

We analysed 123 million bacterial records from GenBank, examining the proportion for which annotation provenance has been recorded or not and whether any linked accessions have since been removed or remain available. We find that 73% (89 million) contain structured information about annotation provenance. Of these records, 10% (9 million) have been annotated based on sequence records which are no longer active, with the linked record having been removed or merged with other records. This estimate of broken links is likely an under-estimate as provenance information for a large proportion of records was ambiguous when no information about sequence version was provided in the inference qualifier.

**Summary:** This case study highlights that for sequence records, at least those in GenBank, the ability to determine the source of an annotation, let alone to propagate corrections from the source, is near impossible. The analysis above only covers a proportion of available records and only considers the most extreme update where the original record was removed. Other types of errors, such as incorrectly recorded provenance or a failure to update annotations when the source record has been altered, will not be captured and could change our understanding of annotation quality. As such, efforts to record annotation provenance need to be improved, both to provide capabilities to store such metadata and to encourage users and developers of annotation pipelines to consistently provide such information.

## Discussion

Analysis of genetic sequences from public databases has become an underpinning methodology for clinical and research work, and thus it is critical that the databases can be trusted. Errors within sequence records have repeatedly been shown to have serious ramifications on downstream analysis, leading to spurious conclusions in analysis and significant loss of time and money. As several papers have demonstrated [13, 17, 78], the rapid growth and limited ability to curate these databases is increasing the amount of erroneous information in these databases over time. While a plethora of tools have been developed to look for various types of errors, most remain limited to a single database or a single class of errors and have not previously been considered as part of a larger framework that considers the high interconnectivity between databases. Perhaps for this reason they have typically had limited impact to date, highlighting the need for alternative approaches for detecting and quantifying errors within biological sequence databases.

We have described how the many types of relationships between biological sequence records can be viewed as a complex network and argue that this network perspective can be used as the basis for the development of methods that detect, track and potentially correct errors both across databases and over time. Moreover, making use of records across databases creates the potential to take advantage of independent curation processes and additional sources of information that are unique to each database. While many methods have been created that draw upon particular kinds of relationships between records, as we describe in Case Study 1, a network perspective allows us to expand the sets of relationships between records that we can draw upon. While our case studies describe several uses of network analysis in the existing literature, there are many further opportunities that could be developed.

### A new broom sweeps clean

Taking a network perspective to sequence records allows us to use a wide range of techniques that have been developed in other network contexts to understand the quality and trustworthiness of aspects of the network. A possibility, discussed in the first case study in this review, is the application of network-based anomaly detection, exploiting the relationships across the sequence database network to extend the existing outlier detection approaches that have already been applied for error detection.

Network-based anomaly detection encompasses a range of techniques to identify records that are different from their local neighbours [67], varying from clique-analysis [79] to joint-matrix factorization [80]. The approach also offers opportunities to incorporate other sources of knowledge, either making use of the existing manual biocuration approaches in databases such as SwissProt, integrating structured ontologies or knowledge from supporting literature itself [81, 82], thus integrating information from PubMed articles with the sequence database network. The flexibility of these network approaches and their ability to combine a mixture of different knowledge sources has the potential to enable powerful new tools to detect records that can be flagged as a suspect for manual inspection.

A network perspective of records in sequence databases can also be used to compute measures of how trustworthy a record is, borrowing trust propagation techniques from machine-learning-based recommender systems [83, 84]. When making automated recommendations for a given user based on behaviour or product ratings from related users, trust propagation seeks to identify which individual’s information can be trusted to be used in the recommendation. Estimates of trustworthiness can also be propagated across multiple relationships, to incorporate nodes in the network that are distant from the given target [83]. Trust propagation can be seen as a more systematic implementation of the metrics proposed by [14], described in the first case study. Rather than considering the distance to a single trustworthy record, trust propagation allows us to summarize the confidence of all previous annotations, providing a flexible framework for integrating background knowledge of annotation confidence. Moreover, as such approaches were designed for systems with many millions of records, they are scalable to the increasingly large number of records in the sequence database network.

### A disease known is half cured

While there is broad agreement that different species or curation processes of different databases will lead to varying quality of sequence records, there are few characterizations of how much quality of sequence records and their annotations vary across species and over time. There are several strong studies of individual types of errors, including duplication [9], contamination [10, 11], protein function [13, 14] and taxonomic misclassification [61–63, 85]. However, only a few have been scaled to entire databases [10, 61, 86], leaving the quality of many records unclear. As Case Studies 2 and 3 both highlight, erroneous and outdated annotations accumulate in the sequence record databases over time, despite the efforts of manual curators and automated tools to detect errors.

While the network perspective proposed in this work offers new potential to detect errors within sequence databases, it can be difficult to ensure that any detected errors are corrected in these databases. This is especially true for INSDC databases where the sequences remain controlled by the original submitter of the sequence, preventing corrections unless the submitter is willing to make an update [33]. While smaller, organism-specific databases [87] have been proposed as a potential solution, it is unclear whether such an approach will scale to encompass all organisms, especially given the limited scalability of manual curation [88].

An alternative possibility is the development of a fully automated database, whereby sequences from INSDC are treated as annotation free and are relabelled from scratch. Multiple annotation pipelines could be used to highlight instances where annotations are consistent (indicating high confidence) or inconsistent (indicating further examination is required). A fully automated would limit the presence of stale annotations and broken links by continually updating the annotations. While TrEMBL partially implements such a solution, it still relies on many of the original annotations, such as taxonomy, and only handles proteins, rather than nucleotide sequences as well.

The disadvantage of such a system would be the limited accuracy of annotations, but a possibility is to ensure that metrics related to confidence and reliability are always available for all annotations. This would allow users to have greater control over the types of records that are used in their experiments, enabling a trade-off between quantity and quality of sequence records that will be application dependent. Moreover, metrics related to confidence could help database curators to understand where available resources should be focused to most effectively improve database quality. Such a database strategy would support the network perspective outlined in this work by providing a rich source of annotations and confidence metrics.

### A chain is as strong as its weakest link

It is easy for errors to propagate across sequence databases. The limited systematic recording of the provenance of record annotations means it is much more difficult to propagate corrections to those errors, with updates to a source record often not reflected in the records that are derived from or depend on that source [15]. An impact consistently highlighted in this review is that the inter-connected nature of sequence records within and across databases means that allowing such errors to persist, will likely lead to further propagation of errors to new records. Propagation of erroneous annotations has been widely discussed [10, 15, 49], with many commentators noting that spurious annotations are extremely challenging to remove from the system given the limited information relating to provenance.

This situation is further complicated as different databases jointly form a single federated database [89], whereby a single sequence can typically be mapped across multiple databases. This introduces challenges in synchronizing updates between different organizations responsible for curation of different databases, and is further complicated by temporal aspects introduced by manual curation processes or database update cycles. Moreover, systematic differences in the recording of provenance information between software pipelines in different databases, in particular changes in accession format, make it challenging to parse annotation links in downstream application.

A necessary precondition for being able to propagate corrections to erroneous annotations is a clear list of all records that the annotation has been propagated to. Without the ability to systematically correct an error in a source record and all of its dependencies, there is a risk that the correctness of public sequence database could degrade over time, with some studies indicating this is already occurring [13, 14, 78]. Our analysis in Case Study 3 indicates that the recording of annotation provenance, at least in bacterial records in GenBank, is more widespread than previously described [15]. Whether these results vary significantly over species and database is an open question for further work. It is clear however that extracting annotation provenance is challenging as recording of this information is only partially standardized and is often stored within free text that cannot trivially be parsed. While it is excellent that some provenance annotations are recorded, improvements to ensure that this information is available in structured formats and accommodates FAIR data principles including interoperability and reusability [90] would allow the community to make better use of this critical information.

The ideal solution is to encourage users and database curators to more systematically record the provenance of annotations, including metrics about the reliability or confidence of these annotations. Such an approach would enable propagation of corrections through databases, enable the development of novel techniques for error detection utilizing these explicit relationships and enable novel metrics capturing database quality. The use of high-level provenance such as the GO IEA evidence code has already been argued to reduce bias in evaluation of automatic prediction methods [91]; more fine-grained provenance would further facilitate assessment of bias or errors in database records. Databases can also improve the recording of annotation provenance by ensuring that any recorded links follow consistent formatting and discouraging the use of storing information within free-text fields. Pipelines developed by NCBI for curation of both prokaryotes [41] and eukaryotes [40] have begun to record greater information about how annotations were derived and which pipelines were utilized, with potential benefits for database quality. This does not address issues in existing records, but the ongoing rapid growth of these sequence databases means that implementing a strong framework for recording provenance is urgently needed.

## Conclusion

Few approaches for error detection or inferring record quality in biological sequence databases take a holistic view of the connectivity of information between records. Our proposed network perspective of sequence databases provides a powerful framework that allows the integration of information across and between both records and databases. Based on these, we highlight the following observations and directions:

- New metrics are needed for quantifying quality and confidence of records and their metadata.
- Inconsistencies between records may be due to failure to propagate updates or corrections; use of links between records may support strategies for achieving improved consistency.
- New methods for detecting annotation errors are needed that draw on the relationships between multiple databases.
- Systematically documenting the provenance of annotations could help reduce issues of inappropriate propagation of annotations.
- Where records can be connected between databases, the connection can be used to verify whether they are correct.
- Propagation of corrections between historical records will remain challenging due to poor documentation of annotation provenance.
- Expansion of ongoing checks of existing records will improve the entire sequence database network ecosystem.

In our view, it is timely to regard the various sequence databases as components of a larger whole rather than as loosely linked independent entities. The potential of this approach can be realized though consistent recording of annotation provenance to improve our recording of inter-record relationships, new methods that exploit the relationships between records and improved practices by record submitters and curators, steps that have costs but which are essential if the quality of sequence data is to be assured.

**Key Points**

- We highlight a perspective on public sequence data, whereby the collection of repositories can be viewed as a single network of interconnected information. This perspective suggests novel extensions to existing methods and highlights a number of novel opportunities.
- The impact of annotation errors due to propagation remains poorly quantified due to limited recording of annotation provenance.
- Novel metrics for quality and confidence of records and their metadata would enable improved selection of sequences for downstream analysis
- Network analysis of multiple linked records, across and within databases, is a promising approach for detecting annotation errors.

## Supplementary Material

2207_Response_to_reviewers_bbac416

## Author Biographies

**Benjamin Goudey** is a Research Fellow at the School of Computing and Information Systems, University of Melbourne. His research interests include genetic epidemiology, genomic risk prediction and machine learning.

**Nicholas Geard** is an Associate Professor at the School of Computing and Information Systems, University of Melbourne. His research areas include computational simulation, complex networks and infectious disease epidemiology.

**Karin Verspoor** is the Executive Dean of the School of Computing Technologies at RMIT University. Her expertise is in artificial intelligence methods to enable biological discovery and clinical decision support from data.

**Justin Zobel** is a Redmond Barry Distinguished Professor in the School of Computing and Information Systems and Pro Vice-Chancellor (Graduate and International Research) at the University of Melbourne.
