# Supplementary material for: Propagation, detection and correction of errors using the sequence database network
Source: Brief Bioinform. 2022 Oct 20;23(6):bbac416. doi: 10.1093/bib/bbac416 (PMC9677457; doi:10.1093/bib/bbac416)
Supplement: 2207_Response_to_reviewers_bbac416 [file 2207_response_to_reviewers_bbac416.pdf]

Response to reviewers

We thank the reviewers for their valuable comments and corrections that have substantially helped the quality of the manuscript.

We have considered all of their suggestions and as discussed in detail below believe that have all been addressed. In particular, we have:

- Clarified our definition of the sequence database network, emphasising that it is richer than explicit parent/child relationships but also includes annotation-based relationships (a mix of explicit and implicit links depending on how much was recorded) and record similarity (including factors such as sequence identity, taxonomic distance, and functional similarity).
- Case Study 1 (previously 3) now links existing methods for error detection in sequence databases with a network-based framework for outlier detection, highlighting the types of links that can be further integrated.
- Case Study 2 (previously 1) now has an empirical analysis of sequence similarity between EC annotations in GenBank and experimentally validated records in SwissProt. We observe that many records seem to have little support, highlighting the utility of simple post-hoc metrics of propagated annotation confidence and how they could be improved using network approaches.
- Case Study 3 (previously 2) now has an empirical analysis of annotation provenance links within the database, how often they are recorded, and how many links are dead. This highlights the need for improved recording of information across the network and for the value of the network perspective.
- Reworked the discussion section to remove repetitive aspects and provide more concrete directions for future research.

Please find below the responses point by point for the comments raised by the reviewers. In addition, we attach a marked version of the manuscript where changes are highlighted in blue for ease of second review and a comment is given in the margin to highlight which of the reviewers comments are being addressed.

1 Reviewer 1

1.1 Major comments

Intro 1 *I am little conflicted about the angle of the review: while there are some really interesting elements, it is not clear to me what the audience might be. The 'novel' idea of a 'sequence database network' needs to be toned down, as it is common practice for database providers who attempt to improve content through cross-referential links, which implicitly represent a network. In other words, for specialists there is little novelty and maybe the 'network' angle might be misunderstood. For a more general audience, this notion needs to be connected with the actual operations of database providers, which are not in fact always very clearly articulated in the literature. Some more clarity and focus might be necessary to improve the perspective provided.*

Response: We appreciate the reviewer's concerns about the degree of novelty in our network perspective and the importance of targeting the correct audience.

While explicit cross-referential links are important, our network perspective also includes links resulting from annotation propagation (sometimes explicit but hard to extract, sometimes implicit) and also sequence, function, and taxonomic similarity (implicit links). Further, we emphasise the kinds of analysis that can be conducted using this analysis, which includes some analyses that are currently conducted but not described in terms of a network. We believe that different aspects of this will be of interest to both general and specialist audiences.

To make this clearer, we have split the section '*An overview of sequence databases*' into two parts. The new section, '*Defining the sequence database network*', explicitly describes our understanding of 'network' and emphasises different kinds of relationship, including both explicit and implicit links between records. These changes begin on page 6, marked as **Rev 1, Intro 1a**.

Concerning the comments about generalists and specialists, in our experience there are few manuscripts that clearly explain the different sequence databases, how they operate, and the impact that their characteristics may have on downstream tasks. While a full description is out of scope, we have added commentary on how these differ. For the specialist, the previous description of the networks between records gave an impression of using simplistic relationships between records, primarily parent/child relationships across databases that we have since expanded. This impression is corrected in the new text.

To address these points, we have:

- Added a paragraph at the end of the section '*An overview of sequence databases*', describing how the different databases have different intentions that are reflected in the

curation processes (page 5, marked as **Rev 1, Intro 1b**).

- Added a sentence at the start of the Discussion section, highlighting how an explicit network perspective extends ideas the field is using and provides scope for integrating more information (page 14, marked as **Rev 1, Intro 1b**).
- Expanded our description of networks in the section '*Defining the sequence database network*', as described above.

Intro 2 *The subtitles in the Discussion section are really cool, and the structure in general is fine.*

Response: We thank the reviewer for their feedback.

Intro 3 *Case studies are a little fuzzy: the authors might consider providing a data supplement for these cases, if possible?*

Response: We agree with the reviewer that the case studies did not have enough detail to fully support the benefits of the network perspective we propose. We have significantly altered the first two case studies, adding exploration of the network on real data and demonstrating its utility. We have also added greater detail to the third case study. Their order in the new manuscript has been also been altered. In particular:

- Case Study 1 (previously 3) now links existing methods for error detection in sequence databases with a network-based framework for outlier detection, highlighting the types of links that can be further integrated (starting page 8, marked as **New Study 1**).
- Case Study 2 (previously 1) now has an empirical analysis of sequence similarity between EC annotations in GenBank and experimentally validated records in SwissProt. We observe that many records seem to have little support, highlighting the utility of simple post-hoc metrics of propagated annotation confidence and how this could be improved using network approaches (page 11-13, marked as **New Study 2**).
- Case Study 3 (previously 2) now has an empirical analysis of annotation provenance links within the database, how much they are recorded and how many links are dead. This highlights the need for improved recording of information across the network (page 13 and 14, marked as **New Study 3**).

1.1 *The situation with sequence databases is a complete mess. Let's start with this presumption. Some of the most important elements that can improve the current chaos have to do with error propagation (many cases exist), phylogenetic inconsistency (unreal sequence assignments to species), metagenomics sequences not belonging to species (and yet assigned to some) and lack of consistency (via wrong or dead links, all over the place). People argued long time ago that -only- experimentally verified sequences need to be annotated with functional elements and the rest should have remained anonymous ("a new sequence in one database may draw*

its annotations…"), a bit too late for this now (see also 1.5). One more concrete idea about "inferences about record quality…" is the stratification of databases at levels of quality. Please comment.

Response: The reviewer's concerns about issues that exist across the sequence databases and how to address them are very relevant to this review. A key challenge is that the extent to which the errors listed above are present across a range of sequence databases remains unclear. While the errors the reviewer has listed are known to exist, it is not clear how frequently they occur.

While addressing all of the types of errors listed above would require multiple pieces of work, we have used the suggestions above to strengthen one of the case studies presented in this review (Case Study 3). In particular, we have:

- Examined the recording of annotation provenance in GenBank (Case Study 3) and whether recorded links are active or dead (page 13, marked as **Rev 1, 1.1a**).
- Discussed the potential for more subtle errors (failure to update, incorrect links) to still remain in Case Study 3 (page 14, marked as **Rev 1, 1.1b**).

1.2 *…growth has outstripped the ability of manual curation", isn't this equivalent to very bad planning? Gets worse, as these are the same people who lecture everybody else about best practices in the field of database maintenance… See also PMID: 12838343. Please comment.*

Response: The reviewer provides an interesting perspective that manual curation was always going to be a challenge to scale as sequencing becomes more widely-used. This perspective underlines why our work is of value, as it highlights the tension between manual and automated curation within the databases. While manual curation is challenging to scale, automated curation remains extremely problematic in many instances; for example, protein function prediction methods are only accurate in around 70% of cases as per CAFA benchmarks.

One approach is to increase automation but simultaneous increase information about the quality of available data. Rather than neglecting the fact that sequence annotations are likely to be incorrect, we believe that the correct approach is to increase and improve the quantification of annotation confidence. These resources are also highly beneficial for network analysis.

To address this point, we have added to the discussion section ('*A disease known is half cured*'), commenting on the limited scalability of manual curation, with the comment made in the context of species-based databases (see reviewer point 1.3). These appear on page 16 and are marked as **Rev 1, 1.2**.

1.3 *Some people argued in the past that sequence annotation with no genomic context is a not such a good idea and that annotation should be happening by species-centric communities, e.g. SGD for yeast, FlyBase for Drosophila etc. See also pangenome-driven annotation efforts. e.g. PMID: 33763039. In our work, we are discovering, with surprise, that certain sequence entries from the very first genomes that have been sequenced remained without any further attention for more than 25 years in SwissProt! Please comment.*

Response: The reviewer raises two interesting points, the first about community-based annotation efforts and the second about record annotations becoming 'stale' over time.

While communities for specific species are likely to provide strong resources and improved annotations for the species they target, it is typically the case that neglected species will always remain, motivating the need for more general curation approaches, whether manual or automated.

The problem of stale annotations across the different databases will continue to grow over time. We believe this motivates fully automated database approaches that would always refresh annotations based on available knowledge.

To address these points, we have added to the discussion section ('*A disease known is half cured*') a commentary on how community-based databases are unlikely to be the main solution to sequence databases and that a fully automated re-annotation of INSDC sequences could be a scalable solution, albeit with different tradeoffs. We have marked these changes on page 15 with **Rev 1, 1.3**.

1.4 *"...most approaches to date do not exploit the highly connected nature of sequence databases": this is connected with the internal operations of database providers, which are not fully and openly documented, as far as we can tell, so it is very difficult for the community to understand the strengths and limitations of these procedures. Please comment.*

Response: The reviewer highlights that full connectivity of records is not entirely available to the public, nor is it clear exactly which relationships are explicitly recorded at all.

From our experience, many of the relationships that exist between records can either be extracted (parent/child, annotation source/target) or derived (sequence or annotation similarity). Some are challenging, with annotation provenance in particular remaining difficult to extract as it is often stored as free text.

We discuss the challenges of extracting information from databases in the discussion section '*A chain is as strong as its weakest link*' and highlight that this is a problem related to data quality and in particular to FAIR principals. We've marked these changes on page 16 as **Rev 1, 1.4**.

1.5 *"The example above highlights that a single sequence record both uses and creates a range of records in other databases" — it would have been much better to keep that sequence free of derived annotations. See also PMID: 27528420. Please comment.*

Response: The reviewer highlights an alternative curation process for INSDC databases, which is that they should allow for sequences to be uploaded and then these sequences should be reannotated.

In some ways, GenBank/INDSC is already used as a source of sequence, though typically databases like RefSeq and TrEMBL assume that annotations such as the source organism are correct. Having a database that produces fully automated annotations of uploaded sequences, potentially using multiple independent pipelines and recording information about annotation confidence, could serve as a further resource to contrast existing annotations and highlight annotations or sequences that could be erroneous. The disadvantage of not allowing submitter to annotate sequences is that potentially critical or unique information would not be made available.

We have added a discussion point in the section '*A disease known is half cured*', describing the potential for a database that applies computationally-inferred annotations on top of INDSC sequences, similar to TrEMBL but covering nucleotide sequences and re-computing even more annotations. Moreover, we propose that metrics related to confidence and reliability could be used in such a database to allow users to understand which annotations are likely to be problematic. These changes occur on page 16 and are marked as **Rev 1, 1.5**.

1.6 *"... large differences in the annotations of genomes resulting from different methods", you bring up a very serious issue here: despite their key role, databases were never properly audited, by the community. Can SwissProt assure us that starting from a single record, their internal procedures applied independently will result in the same metadata? I strongly doubt it, never been done. Maybe they are doing this internally, but we do not know. Please comment.*

Response: The reviewer highlights that the manual curation processes are subjective to some extent and that this is not well documented.

Given the strong role of manual curation in the annotation process for UniProt, it is likely that different annotators produce different sets of metadata for the same record. This has been observed with GO annotation of medical literature (PMID 25070993), with high inter-annotator disagreement observed in particular instances. This disagreement is likely to be higher when looking at sequences directly.

To address this point, we have added to the section '*Annotation errors in biological sequence records*', highlighting that different curation pipelines, including both different tools or different curators, will lead to conflicting outcomes which can introduce errors. These changes

occur on page 7 and are marked as **Rev 1, 1.6**.

1.7 *"relies on two assumptions": correct, and importantly, without strict criteria of admitting a target to the source annotations (mentioned in "limits to how far annotations should be transferred", what are those limits? — we don't really know). In UniProt, we have seen a great variation in applying (unknown) criteria. Also, we note that 'easy' (and not very useful) entries are progressing with annotation, the tricky (and most interesting) entries do not. Please comment.*

Response: The reviewer highlights that its often unclear how far annotations in existing curation processes are allowed to propagate (what are the constraints around similarity).

This points aligns very well with the case study that considers the work by Rembreza and Enqvist (2020) as well as the experimental evidence we've added in the latest revision. Both sets of results highlight that over-propagation frequently occurs, at least in enzymes, and this likely leads to many erroneously annotated sequences.

To further emphasise these points, we have added to the section '*Annotation errors in biological sequence records*', highlighting that the limits as to how far annotation vary by sequence, species and function. These occur on page 7 and 8 and have been marked as **Rev 1, 1.7**.

The comment made in relation to 1.6 about inter-annotator disagreement also supports this discussion point.

1.8 *"A network perspective of sequence records may offer novel approaches": connected to the above, I would rephrase as "A network perspective of sequence records explicitly provided by database providers with an appropriate technological framework may offer...", or similar. Please comment.*

Response: The reviewer notes that our description in the submitted manuscript primarily refers to explicitly recorded relationships.

In the initial version of the manuscript, we emphasised cross-databases relationships where one record leads to the creation of a record in another. However, there are a wide range of relationships that are explicit but hard to extract (annotation) or are implicit (similarity). Moreover, as we describe throughout the paper, there are existing case studies that have made use of network analyses cross-database information about sequence to detect errors or understand database quality. The critical insight is that these approaches use the network implicitly. By explicitly re-framing these approaches as network-based, we can view these approaches in a more general framework and can more easily see opportunities to expand and improve these approaches.

We have altered the relevant sentence to this effect at the end of the section '*Annotation errors in biological sequence records*'. These occur on page 8 and are marked as **Rev 1, 1.8**.

1.9 *Table 1 does not attempt to prioritize the 'seriousness' of these errors, see also PMID: 11864365, table 2. Please comment.*

<u>**Response:**</u> The reviewer correctly identifies that Table 1 presents common errors but we have not ranked them by severity.

Our intention of this table was to provide examples to the general audience of the types of errors that can occur in sequence records, explicitly calling out incorrect propagation as a class of error that is typically ignored.

To address the reviewer's point we have added a statement referencing this paper (PMID: 11864365) in the section *'Annotation errors in biological sequence records'*. These occur on the middle of page 8 and are marked as **Rev 1, 1.9**.

1.10 *"We have proposed a network perspective of biological sequence databases", again, it might be wiser to state that the authors emphasize the value of a broader network-based perspective, which is fine, instead of claiming novelty — just so that they do not upset database providers..*

<u>**Response:**</u> The reviewer's point, that we are not the first to have considered a network perspective is a reasonable one. However, while there have been others who have used network analyses of sequence databases, these are almost always implicit. Explicit use of network analyses remains very under-explored.

We have adjusted the wording of the relevant sentence to state 'We have described how the many types of relationships between biological sequence records can be viewed as a complex network', that is that we are emphasising a network perspective as being valuable, rather than indicating we are the first to consider this idea. The change is on page 14, marked as **Rev 1, 1.10**.

1.11 *"further complicated by challenges in communicating updates", also worth mentioning the differential generated by legacy software technology in various settings, although there are new technologies that can help here. Not all problems can be solved by technology alone, yet I think suggesting a more coordinated effort with some underlying technological frameworks can be of value.*

<u>**Response:**</u> We are unclear as to whether the reviewer is highlighting that different databases make use of different curation pipelines or whether different databases are underpinned by different technologies to manage records and updates.

We assume they refer to curation pipelines as we have little information about how releases between databases are managed at a technology level. These curation pipelines are quite opaque, even when the software itself is available. However, it is clear that differences in curation pipelines across databases introduce differences in annotations when given exactly the same underlying sequence.

We have addressed this comment in two locations:

- In the first paragraph of the discussion section '*A chain is as strong as its weakest link*' we have added a sentence on the use of different annotation pipelines recording annotation provenance in different ways. These are marked as **Rev 1, 1.11a** on page 16.
- In the second paragraph of the same discussion section, we have commented that databases should have greater enforcing of accession reporting standards to help parsing of records. These are marked as **Rev 1, 1.11b** on page 17.

1.12 *"... encourage users and database curators", raises an important point, related to funding. IMHO, it is totally unacceptable for projects such as SwissProt to contain trivial errors after 100s of millions of euro-dollars. Who is going to provide incentives to users? Perhaps some sort of gamification of annotation updates by community members could be a solution. Please comment.*

Response: The reviewer raises the point related to funding of sequence databases.

While an important point, we have decided to not comment in the article on this topic, as funding arrangements for these databases is out of scope. Likewise, commenting on the motivations of users is challenging given that many users are motivated within the context of specific species databases and approach these databases for a diverse range of tasks.

1.2 Minor comments

2.1 *"An underappreciated property of these sequence databases is their strong interdependency", this may not be true. Better say something like: a property of these databases that can be used to improve the situation is their strong interdependency?.. Something like that. Same for "novel perspective", perhaps better say something like "describe the network perspective as a means to continue improving quality and content of sequence databases"...*

Response: We have altered this statement in the abstract as suggested, making sure to emphasise a broad range of relationships between records. stating now 'One property of these sequence databases that can be used to detect such errors are the strong interdependency between records.' These changes are marked on page 1 as **Rev 1, 2.1**.

2.2 *"provide a novel classification of sources of error" in abstract, please see 1.9.*

Response: We have removed the term 'novel' here, given the overlap with the reference given in 1.9. These changes are marked on page 1 as **Rev 1, 2.2**.

2.3 *ref [7] a bit obscure? there are better ones...*

Response: We have altered the reference to point to

- Yandell, M. and Ence, D., 2012. A beginner's guide to eukaryotic genome annotation. Nature Reviews Genetics, 13(5), pp.329-342
- Richardson, E.J. and Watson, M., 2013. The automatic annotation of bacterial genomes. Briefings in bioinformatics, 14(1), pp.1-12.

The relevant change now occurs on page 2, marked as **Rev 1, 2.3**

2.4 *"Any identified proteins will indexed" will -be- indexed.*

Response: This has been corrected. The change appears on page 3, marked as **Rev 1, 2.4**.

2.5 *"The example above highlights that a single sequence record both uses and creates a range of records in other databases", a good reference here might be PMID: 8987457, a more recent one might be PMID: 19060306.*

Response: We thank the reviewer for the suggestion. We have added the reference to PMID: 8987457. However, it does not strongly support the argument here directly, as the focus was largely on representation of provenance information. The additional reference has been added on page 3 and is marked as **Rev 1, 2.5**.

2.6 *"...sequences remain owned by submitters", this is another huge issue that prevents corrections at source, see also PMID: 30679363 and 18356505. Needs to be discussed briefly.*

Response: We thank the reviewer for their suggested references about sequence ownership. We agree that this is an important topic but there is limited scope within this manuscript to highlight all of the design choices that have been made across the collection of sequence databases.

We do agree though that a reference to a discussion of this topic is needed. To this end, we have added a reference to 18356505, which is very relevant, but not to 30679363, which seems to focus on different kinds of issues with data sharing. We have added this in two places, the first in the area the reviewer identified (page 4, marked as **Rev 1, 2.6**) and the second in the discussion (page 15, marked as **Rev 1, 2.6**).

2.7 *"A subset of high quality, non-redundant sequences are then reannotated and stored in the RefSeq database", a subset which is neither high quality nor non-redundant...*

Response: We have changed this to be a quotation taken from the RefSeq paper to indicate that this is the intention. However, we are not aware of any study that has examined whether RefSeq contains low quality or redundant sequences (two overloaded terms that authors have found hard to define).

2.8 *"including Pfam, SMART, TIGRFAMs, PANTHER, and CDD", you will need original references for those.*

Response: These references have since been added.

2.9 *"Moreover, the curation strategies", see also 1.4.*

Response: The reviewer is presumably highlighting that it is difficult to comment on the strategies of different databases given the limited documentation of internal strategies (as per comment 1.4). We agree, but in this instance our comment is only meant to highlight that databases vary in their degree of automation and their update cycles. As such, we believe the existing text is reasonable.

2.10 *"propagation-based approaches [7, 35, 36]", are you sure you want to use ref. [36]? a bit basic. Maybe PMID: 19226438.*

Response: We have updated the citations.

2.11 *"they have the potential to be propagated to new records", maybe a reference here, PMID: 12490449.*

2.12 *"... there are fewer studies that explicitly focus on detecting errors stemming from incorrect propagation...": case studies that assess propagation for specific protein families are available, e.g. PMID: 20011109 (cited in Case study 3, it might also belong to Case study 1, as citation for similar work), same for genome-scale annotations, e.g. PMID: 29806194.*

2.13 *"quantifying the confidence in propagated annotations as an indicator of potential error", see PMID: 16354297 and PMID: 17570146.*

2.14 *"many tools implicitly making use of cross-database information", perhaps also cite other (better) work, PMID: 25653249.*

Response: We have added the suggested citations for points 2.11-2.14.

2.15 *"errors may go uncorrect", uncorrected?*

Response: This has been corrected to 'uncorrected'.

2.16 *"has been enter", entered?*

Response: This has been corrected.

2 Reviewer 2

2.1 Major comments

1.1 *Database primary sources are usually same, so the claimed propagation of effects to secondary databases may be same, not compounded as claimed. I don't think tertiary databases will use the protein information from secondary databases while primary ones are equally accessible.*

Response: The reviewer makes the point that that errors are unlikely to compound over time as errors flow only from primary sources to secondary databases.

This could be true if we are only considering parent-child relationships, which are regularly updated across the different databases. If a source record is removed, all of its child records are typically also removed (we note, though, that the UniProt documentation admits this does not always occur). However, if we consider other types of relationships, and in particular propagation of annotations, there are currently no update mechanisms to ensure that these annotations are corrected over time. Moreover, annotations from secondary databases such as RefSeq and UniProt are used to annotate records in INSDC databases such as GenBank, creating the possibility of errors becoming compounded.

To highlight this issue, we expanded case study 2 '*Interrogating the network to estimate the reliability of annotations*' to show the number of annotations that are derived from records that have since been removed. If these source records were removed because of errors, its possible that these errors remain reflected in these records that make use of their annotations. These changes are marked on page 14 as **Rev 2, 1.1**.

1.2a *The manuscript text has nitpicking cases that do not clarify the issue properly, so without the desired effect on reader. A figure might help summarize the issues in a better way.*

Response: We have substantially changed the three case studies in this manuscript to better highlight the impact of network analysis. In particular:

- Case Study 1 (previously 3) now links existing methods for error detection in sequence databases with a network-based framework for outlier detection, highlighting the types of links that can be further integrated (starting page 9, marked as **New Study 1**).
- Case Study 2 (previously 1) now has an empirical analysis of sequence similarity between EC annotations in GenBank and experimentally validated records in SwissProt. We observe that many records seem to have little support, highlighting the utility of simple post-hoc metrics of propagated annotation confidence and how this could be improved using network approaches (page 10 and 11, marked as **New Study 2**).
- Case Study 3 (previously 2) now has an empirical analysis of annotation provenance links within the database, how much they are recorded and how many links are dead. This highlights the need for improved recording of information across the network (page 12-14, marked as **New Study 3**).

1.2b *The current figure (1) is not clear to me. Why are there slanted lines and horizontal lines to represent sequences? The colored arrows in legend can't be seen in fig. It can be clarified in legend.*

Response: The reviewer provides useful insights into aspects of Figure 1 that were unclear or undocumented. The angles and colours were meant to provide a distinction for the reader between different types and instances of sequences. We agree that greater explanation would help with this figure.

To address this, we have extended the caption for Figure 1 to ensure that the figure can be read more easily, ensuring that all features of the plot have been explained. These changes can be found in the caption for Figure 1 on page 2.

1.3. *Also, systematic ways to tackle database error propagation were not discussed in detail. A superficial reference to network approach without how it can be practically implemented and where (these are federated database cases) it can be performed, makes it a weak or abstract solution.*

Response: The reviewer indicates that more could be written about how to tackle database error propagation, incorporating comments about how the sequences databases collectively form a federated database.

The way to tackle database error propagation is to remove errors in all affected records when an error is uncovered. This requires systematic recording of annotation provenance, i.e where and when did an annotation given to a record arise from. After that, the correct correction strategy is re-annotation of all affected records. The manuscript now covers this topic in substantial detail.

To address the reviewer's points, we have:

- Expanded Case Study 3, which now examines how well annotation provenance is recorded in bacterial records in GenBank, examining 123 million records in a newly added experiment, showing that 73% of records have some provenance information recorded. These have been marked as **New Study 3**, starting page 13.
- Altered, the third discussion section, highlighting that annotation needs to be consistently recorded, but also needs to be recorded in a computationally readable format that adheres to FAIR principals. These have been marked as **Rev 2, 1.3a** on page 16.

In our view, the different databases do not form a federated database as usually understood. While there are consistent interfaces that link across the databases (such as that provided by NCBI), the contents of the component systems is a single database that has integrated information from multiple sources. In contrast, most federated databases provide a unified interface but query multiple databases on the back end. Articulating these differences is challenging given a lack of information about the internal systems of the different databases and is out of scope for this manuscript.

Nevertheless, we have added a brief link to the concept at the start of the third discussion point, marked as **Rev 2, 1.3b** on page 16.

1.4. *Many databases that sync with primary sources are periodically updated and thus will easily correct errors, upon next update. The cycles are fairly regular (like few months) and wont propagate errors too far as the links are usually one to one. If not, you cannot propose to see them as highly interconnected network. The neglected error idea seems counterintuitive.*

Response: The reviewer notes that the description of a network in the manuscript, emphasising primarily parent/child relationships, was either lacking errors or was uninformative.

After taking on reviewer feedback, we believe that our original description of the network was too narrow. Our network perspective natural makes use of a wide range of relationships between records including links through annotation propagation (sometimes explicit but hard to extract, sometimes implicit) and sequence, function and taxonomic similarity (implicit links). This was not as clearly reflected in the original text as we would have liked.

To clarify our definitions, we have split the section '*An overview of sequence databases*' into two parts. The new section, '*Defining the sequence database network*', explicitly describes a network and emphasises that there are different kinds of relationships, including both explicit and implicit links between records. These changes begin on page 6, marked as **Rev 2, 1.4**.

1.5. *Network perspective already exists due to interconnections and opportunities already exist for corrections. A figure can be helpful to explain this.*

Response: The reviewer highlights that a network is a natural way to view existing records.

This perspective motivates our work. However, we feel that while many researchers have made use of the network at one level, explicitly calling out the existence of this network and the ability to run network-based algorithms has been under-explored in this context.

To address these points, we have added a figure and further text to Case Study 1, showing how a network perspective can help in error detection based on outlier detection. The additional figure is marked as **Rev 2, 1.5** on page 8.

1.6. *Strange headings in discussion did not help accentuate the point, rather felt like more repetition. Can be merged or summarized in better way.*

Response: Its unclear to us whether the reviewer dislikes the headings in the discussion section or finds the content to be too repetitive. While headings are a matter of taste (and Reviewer 1 found them to be appealing), we have adjusted the content to ensure that there is greater clarity to the points being made.

We have clarified the discussion points so that the three points cover:

1. Opportunities for novel network based analyses and techniques.
2. Alternative strategies for curating sequences database, in a manner that would align well with the network properties of the underlying data.
3. Improvements in the recording of annotation confidence and provenance.

In doing so, we have reduced the apparent repetition amongst these sections.

1.7. *The Case studies do not provide justice to the title. They could be elaborate and clear examples but I could not understand the take home message.*

Response: We agree with the reviewer that the case studies did not have enough detail to fully support the benefits of the network perspective we propose. This also seems to be the same as the point in 1.2, and we address the point fully there, noting that we have substantially modified the three case studies in this work.

1.8. *Table 1 can have another column to mention the ways/method to catch that type of error?*

Response: We agree with the reviewer that describing the methods currently used to detect errors is useful. However, we regard this is out of scope of the paper and it would require a significant expansion of the text. We have addressed the reviewer's point to some extent by adding a column marked 'References' to Table 1 with references to relevant tools.

1.9. *Authors mention that there are plethora of tools to catch such errors – a table of such tools would add better value.*

Response: The author raises the reasonable point that going into more depth about tools to detect errors in databases would be useful. While this is true, similar to comment 1.8, we find this to out of scope for the current document. The set of tools is quite wide and few are explicitly based on a network approach.

To partially address this, Case Study 1 now describes a range of existing methods in a general framework for outlier detection and highlights that this can be viewed as a network approach. The relevant paragraph appears on page 8 and 9 and has been marked as **Rev 2, 1.9**.

2.2 Minor Comments:

2.1. *Figures in text are without the number but with "??" marks*

Response: This was related to formatting of the document during submission and should now be resolved.

2.2. *Abbreviations need expansion – INDSC, GTDB in fig 2*

Response: This has been corrected.

2.3. *Network perspective is inherent by design due to the database interconnectedness. What are feedback loops ?*

Response: Feedback loops here are caused by new sequences being annotated by existing records. We have altered the wording of the paragraph to try and clarify this. The relevant change occurs on page 5 and has been marked as **Rev 2, 2.3**.

2.4. *Re-use of annotations means?*

Response: We have changed this to *"propagation of annotations.* The relevant change occurs on page 7 and has been marked as **Rev 2, 2.4**.

2.5. *Uniprot has gold, silver and bronze level annotations and also Protein evidence levels 1-5 incorporated into their database. This is ignored and claimed otherwise.*

Response: The reviewer highlights that levels of evidence do exist for some data types, at least in UniProt.

This is correct but only provides information on a fraction of annotations. In particular, the 1-5 evidence levels are whether the protein exists and are not to be used for evidence of the annotations. We have been unable to find evidence about gold, silver, and bronze annotations. However, there is a distinction between manual and automated (colored gold and blue respectively).

2.6. *Page 7, case study 2, para 1, change the line to "...go uncorrected and hence..."*

Response: This has been corrected. We have marked the change now on page 13 as **Rev 2, 2.6**.

2.7. *Page 8 line 1, change to " ...that has been entered...".*

Response: This has been corrected. We have marked the change now on page 13 as **Rev 2, 2.6**.

2.8. *Page 9, second last line change to "...propagation of the error to new records...".*

Response: This has been corrected.

3 Reviewer 3

1 *The authors need to be more concrete about the analyses that will come about from taking a network view of sequence database entries and how they will be enabled by the improved documentation of provenance and quality metrics they call for. The only specific analysis proposal I read is in the Discussion-A new broom sweeps clean. They propose that trust networks could improve on the sequence similarity approach used in Rembeza and Engqvist (2021). In Case study*

1. they indicate that new analyses would be enabled along the lines of Rembeza and Engqvist (2021) if more systematic recording of quality annotations were implemented. But the reader is left to wonder about whether the trust network approach could be implemented with current data or if the proposed systematic quality annotations are required. It would be helpful for the authors to close the circle and make a more concrete claim about how improved annotation would enable the specific analysis they propose.

Response: The reviewer highlights the need to give more specific examples for the case studies.

We agree with the reviewer that the case studies did not have enough detail to fully support the benefits of the network perspective we propose.

We have significantly altered the first two case studies, adding some exploration of the network in real data and demonstrating its utility. We have also added greater detail to the third case study. Their order in the new manuscript has been also been altered, as discussed above.

2 *Similarly, in case study 2 the authors give an example of how an innocuous error propogates through the network. But it's not clear how emergent properties of the sequence database network are expected to advance error propagation analysis or how that network perspective critically requires new annotation provenance data more than individual parent-child relationships. I don't see how the new network perspective increases the need for annotation data relative to an individual record perspective.*

Response: The reviewer raises two issues, the first that case study 2 was insufficiently clear, the second that it is unclear why more provenance information was needed if the network is largely parent/child relationships.

The first concern is addressed in the previous point (point 1) and we have addressed it by substantially extending case study 1.

The second concern, about the limited motivation for annotation data, was in part due to our limited characterisation of the sequence network. Our emphasis in the first submission was overly focused on parent/child relationships, which are given as explicit links in the different sequence databases. However, much of the advantage of our network perspective comes from integrating a wide range of other relationships

While explicit cross-referential links are important, our network perspective also includes links through annotation propagation (sometimes explicit but hard to extract, sometimes implicit) and sequence, function and taxonomic similarity (implicit links). We also emphasise the types of analysis that can be conducted using this analysis, which includes some analysis currently conducted but not thought of as network analyses. We believe that different parts of this will be of interest to both general and specialist audiences.

To make this clearer, we have split the section '*An overview of sequence databases*' into two parts. The new section, '*Defining the sequence database network*', explicitly describes what we mean when discussing a network, making sure to emphasise different types of relationships, including both explicit and implicit links between records. These changes begin on page 6, marked as **Rev 3, 2**.
